# Everolimus and plicamycin specifically target chemoresistant colorectal cancer cells of the CMS4 subtype

**DOI:** 10.1038/s41419-021-04270-x

**Published:** 2021-10-21

**Authors:** Jiayin Deng, Ai-Ling Tian, Hui Pan, Allan Sauvat, Marion Leduc, Peng Liu, Liwei Zhao, Shuai Zhang, Hui Chen, Valérie Taly, Pierre Laurent-Puig, Laura Senovilla, Yingqiu Li, Guido Kroemer, Oliver Kepp

**Affiliations:** 1 MOE Key Laboratory of Gene Function and Regulation, State Key Laboratory of Biocontrol, School of Life Sciences, Sun Yat-sen University, Guangzhou, China; 2 Université Paris Sud, Paris Saclay, Faculty of Medicine, Kremlin Bicêtre, France; 3 Metabolomics and Cell Biology Platforms, Gustave Roussy Cancer Center, Université Paris Saclay, Villejuif, France; 4 Centre de Recherche des Cordeliers, Equipe labellisée par la Ligue contre le cancer, Université de Paris, Sorbonne Université, Inserm U1138 and CNRS SNC 5096, Institut Universitaire de France, Paris, France; 5 Unidad de Excelencia Instituto de Biología y Genética Molecular (IBGM), Universidad de Valladolid – CSIC, Valladolid, Spain; 6 Pôle de Biologie, Institut du Cancer Paris Carpem, APHP, Hôpital Européen Georges Pompidou, Paris, France; 7 Suzhou Institute for Systems Medicine, Chinese Academy of Medical Sciences, Suzhou, Jiangsu China; 8 Karolinska Institutet, Department of Women’s and Children’s Health, Karolinska University Hospital, Stockholm, Sweden

**Keywords:** Cancer therapy, Apoptosis

## Abstract

Colorectal cancers (CRC) can be classified into four consensus molecular subtypes (CMS), among which CMS1 has the best prognosis, contrasting with CMS4 that has the worst outcome. CMS4 CRC is notoriously resistant against therapeutic interventions, as demonstrated by preclinical studies and retrospective clinical observations. Here, we report the finding that two clinically employed agents, everolimus (EVE) and plicamycin (PLI), efficiently target the prototypic CMS4 cell line MDST8. As compared to the prototypic CMS1 cell line LoVo, MDST8 cells treated with EVE or PLI demonstrated stronger cytostatic and cytotoxic effects, increased signs of apoptosis and autophagy, as well as a more pronounced inhibition of DNA-to-RNA transcription and RNA-to-protein translation. Moreover, nontoxic doses of EVE and PLI induced the shrinkage of MDST8 tumors in mice, yet had only minor tumor growth-reducing effects on LoVo tumors. Altogether, these results suggest that EVE and PLI should be evaluated for their clinical activity against CMS4 CRC.

## Introduction

Colorectal cancer (CRC) represents a continuous therapeutic challenge calling for personalized approaches that are based on molecular stratification systems. Thus, beyond the tumor-node metastasis (TNM) classification of CRC stages, anatomical criteria (right versus left, colonic versus rectal cancer), and histological evaluation (low-grade versus high-grade), additional variables have been used to distinguish different categories of CRC [1, 2]. For instance, CRC has been classified as a function of the activated oncogenes (e.g., KRAS-positive versus KRAS-negative CRC) [3], as a function of the immune infiltrates (the immunoscore reflecting the density of CD3^+^ and CD8^+^ T cells) [4, 5] or as a function of microsatellite instability (MSI) resulting from DNA mismatch repair (MMR) defects [6]. All these classifications have clinical utility as exemplified by the fact that KRAS-positive CRC are resistant against the anti-epidermal growth factor receptor (anti-EGFR) antibody cetuximab [7, 8], immunoscore-positive resectable CRC have an intrinsically good prognosis and can be spared adjuvant chemotherapy [9, 10], and MMR-deficient, MSI-high cancers are particularly susceptible to immunotherapy with the PD-1-blocking antibody nivolumab [11–13].

In a collective attempt to unify distinct classification systems, the CRC subtyping consortium identified four consensus molecular subtypes (CMS): CMS1 (microsatellite instability immune), CMS2 (canonical), CMS3 (metabolic), and CMS4 (mesenchymal) [14]. Among the subtypes, CMS1, which is characterized by genomic and chromosomal instability and strong immune infiltration, has a particularly good prognosis [15], while the CMS4 subtype has a particularly poor prognosis, which may be explained by cancer cell-intrinsic features reflecting epithelial–mesenchymal transition and dedifferentiation [16, 17].

Of note, the susceptibility of distinct CRCs to anticancer drugs correlates with the CMS classification, as determined in primary colorectal cancers, cell lines, and patient-derived xenografts [18, 19], as well as retrospective clinical studies [20, 21]. Based on the observation that CMS4 cells are particularly resistant against chemotherapeutic interventions, we employed high-throughput screening to identify drugs that selectively act on such cells. Here, we report that everolimus (EVE) and plicamycin (PLI) are particularly efficient against a CMS4 cell line in preclinical experiments.

## Results

### Identification of everolimus and plicamycin as CMS4-targeting agents

**Fig. 1:**
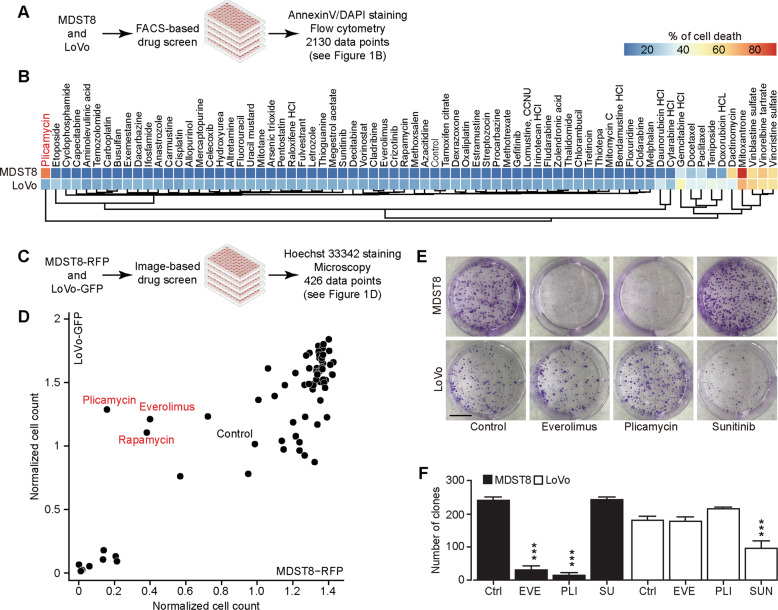
Chemical compound screen discovers that plicamycin and everolimus specifically target MDST8. **A** Scheme of the screening campaigns. **B** MDST8 or LoVo cells were treated with 71 drugs in the anticancer library at a concentration of 0.1 μM for 72 h. The percentage of AnnexinV^high^DAPI^high^ cells was measured by flow cytometry as an indicator for cell death. Each parameter depicts the mean value of three times repeated experiments and is depicted in a hierarchically clustered heatmap. The blue and red tiles in the heatmap represent the percentage of Annexin V^high^DAPI^high^ death cells range from 0 to 100%. **C** Identification of plicamycin, everolimus, and rapamycin as chemicals that specifically kill MDST8 but not LoVo cells. MDST8-RFP or LoVo-GFP cells were treated with 71 drugs in the anticancer library at the concentration of 0.1 μM for 72 h. Debris and cells depicting nuclear pyknosis were excluded, and healthy cells were enumerated. The untreated control was normalized to 1. **D** Results reported in a bi-parametric plot, showing the normalized healthy cell counts after treatment comparing between MDST8-RFP and LoVo-GFP. **E** Images show representative pictures of colonies formed as observed upon crystal violet staining after treatment of MDST8 and LoVo cells with 10 nM everolimus (EVE), 10 nM plicamycin (PLI), or 2 μM sunitinib (SUN) for 3 to 4 weeks. **F** The bar chart represents the number of clones with a size greater than or equal to 50 μm^2^. Error bars indicate SEM. Asterisks refer to significant effects for treatments versus control (Ctrl) (paired Student’s *t* test, ***P < 0.001).

LoVo cells represent the good-prognosis microsatellite instable-enriched CMS1 CRC subtype, while MDST8 cells represent the poor-prognosis mesenchymal CMS4 CRC subtype, as determined by transcriptomic analyses [19] and validated by quantitative reverse transcriptase-polymerase chain reactions (qRT-PCR) for a selected panel of mRNAs (Supplementary Fig. S1). Since CMS4 tumors have a poor prognosis [14, 22] and CMS4 cells are notoriously resistant to chemotherapeutic drugs [19], we designed a dual-screening campaign for identifying drugs that would kill MDST8 cells more efficiently than LoVo cells. In the first approach, both cell lines were cultured in the presence of a collection of ~70 distinct small-molecule anticancer drugs, and the frequency of apoptotic or necrotic cells was determined by Annexin V-AF647/DAPI staining, considering both Annexin V-AF647^+^DAPI^−^ and Annexin V-AF647^+^DAPI^+^ cells as a desirable outcome (Fig. 1A, B and Supplementary Fig. S2A, B). In the second approach, LoVo cells were stably transduced with green fluorescent protein (GFP) and MDST8 cells with red-fluorescent protein (RFP), cultured in the presence of the drugs, and then subjected to automated quantification of the proportion of green and red cells in each culture (Fig. 1C, D and Supplementary Fig. S2C). Both approaches revealed that MDST8 cells were generally more resistant against anticancer drugs, in accord with the published literature [19], with the notable exception of plicamycin (PLI), which was identified in both screens as an MDST8-specific drug, and two inhibitors of the mechanistic target of rapamycin complex 1 (mTORC1), rapamycin and everolimus (EVE), which were identified in the second screen. As a note, the tyrosine kinase inhibitors sunitinib (SUN) and crizotinib (CRIZ) preferentially killed LoVo cells but not MDST8 cells (Fig. 1E, F and Supplementary Fig. S2A, B). Clonogenic assays (Fig. 1E, F) confirmed that both PLI and EVE reduced the number of viable colonies of MDST8 but not of LoVo cells. Hence, we decided to continue the characterization of these two agents, EVE and PLI, as potential CMS4-targeting agents.

### Selective induction of MDST8 cell stress and death by everolimus and plicamycin

**Fig. 2:**
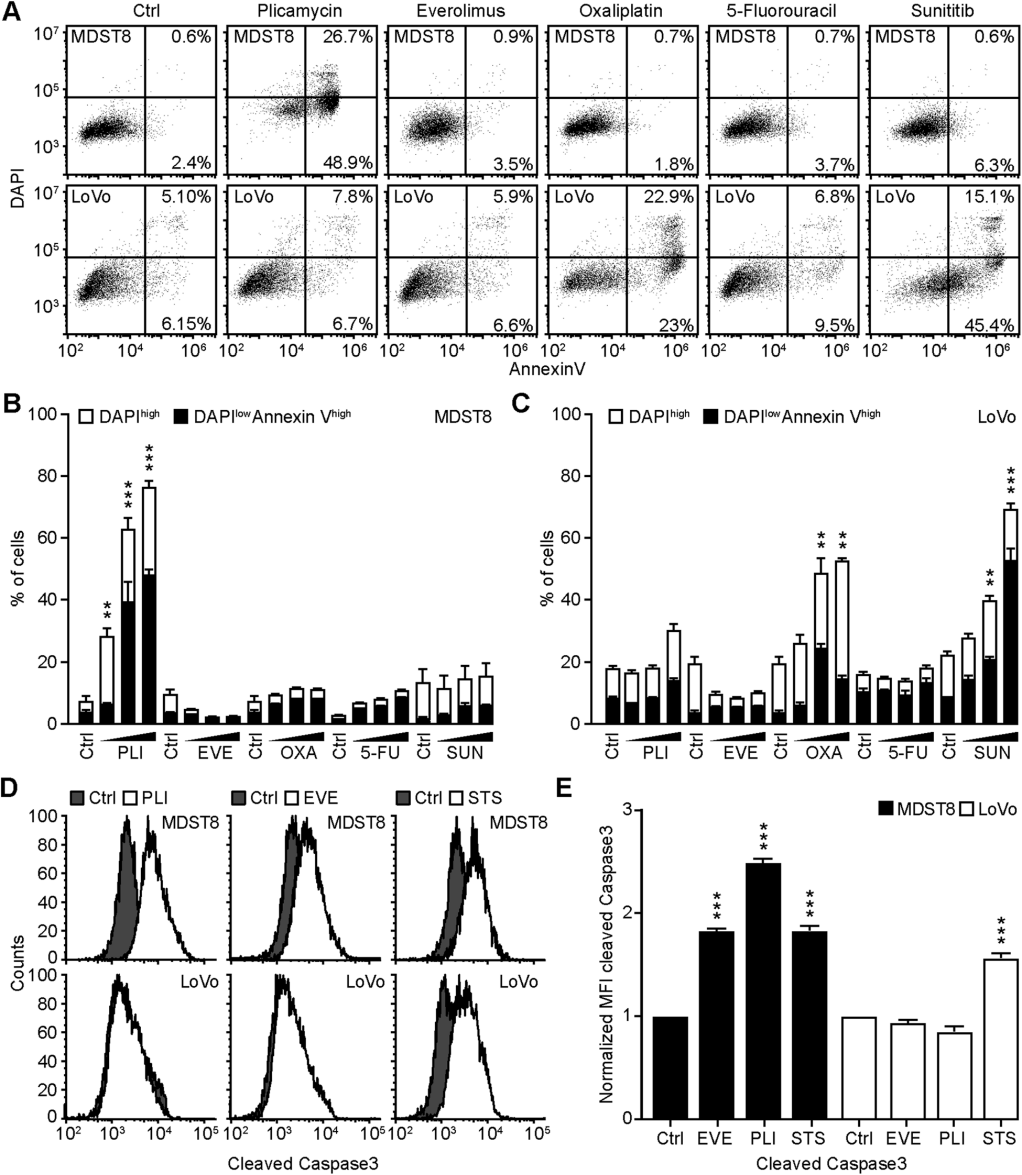
Plicamycin induces cell death in MDST8. Wild-type (WT) MDST8 and LoVo cells were treated with plicamycin (PLI at 25, 50, and 100 nM for 72 h), everolimus (EVE at 10, 100 nM and 1 μM for 72 h), oxaliplatin (OXA; 2.5, 5 and 10 μM for 48 h), 5-fluorouracil (5-FU; 2.5, 5 and 10 μM for 48 h), sunitinib (SUN; 2.5, 5 and 10 μM for 48 h). Then, cells were stained with the DAPI and Annexin V to measure apoptotic cell death (**A**–**C**). **A** Representative dot plots of untreated MDST8 and LoVo controls (Ctrl) or treated with plicamycin 100 nM, EVE 1 μM, OXA 10 μM, 5-FU 10 μM, and SUN 10 μM. Numbers indicate the percentage of cells in each quadrant. **B**, **C** The frequency of dying (DAPI^low^AnnexinV^high^) and dead (DAPI^high^) cells among the MDST8 (**B**) and LoVo (**C**) cells elicited by the corresponding drugs, as determined by analysis with the FlowJo software. Data are depicted as mean values of three independent experiments. **D**, **E** MDST8 cells were treated with 50 nM PLI, 0.1 μM EVE or the positive control staurosporine (STS) 0.1 μM for 48 h. Caspase-3 activation was measured by flow cytometric analysis upon staining with specific antibodies. Representative histograms are shown in (**D**). Normalized mean fluorescent intensity (MFI) of cleaved caspase-3 for each condition is depicted as bar chart (**E**). Error bars indicate SEM. Asterisks refer to significant effects for treatments versus control (paired Student’s *t* test; **P < 0.01, ***P < 0.001).

**Fig. 3:**
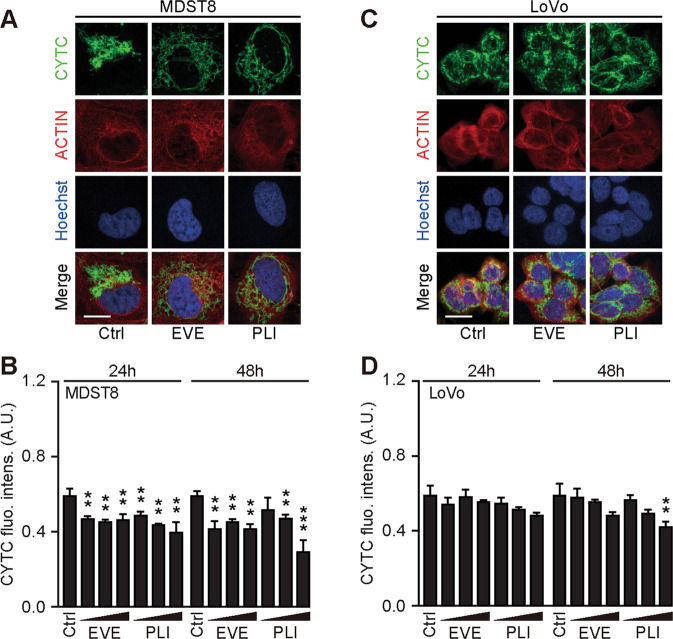
Mitochondrial cytochrome *c* release in response to plicamycin treatment. Wild-type (WT) MDST8 and LoVo cells were treated 25, 50, or 100 nM plicamycin (PLI) or 10 nM, 100 nM, 1 µM everolimus (EVE) for 24 h or 48 h followed by immunofluorescence staining with antibodies specific for cytochrome *c* and subsequent assessment by confocal microscopy. Representative images of cells in each condition are shown. Scale bars represent 10 μm. **A**, **C** Images were quantified of cytoplasmic cytochrome *c* intensity and are reported as a bar chart (**B**, **D**). Error bars indicate SEM. Asterisks refer to significant effects for treatments versus control (paired Student’s t test Error bars indicate SEM. Asterisks refer to significant effects for treatments versus control (paired Student’s t test; **P < 0.01, ***P < 0.001).

**Fig. 4:**
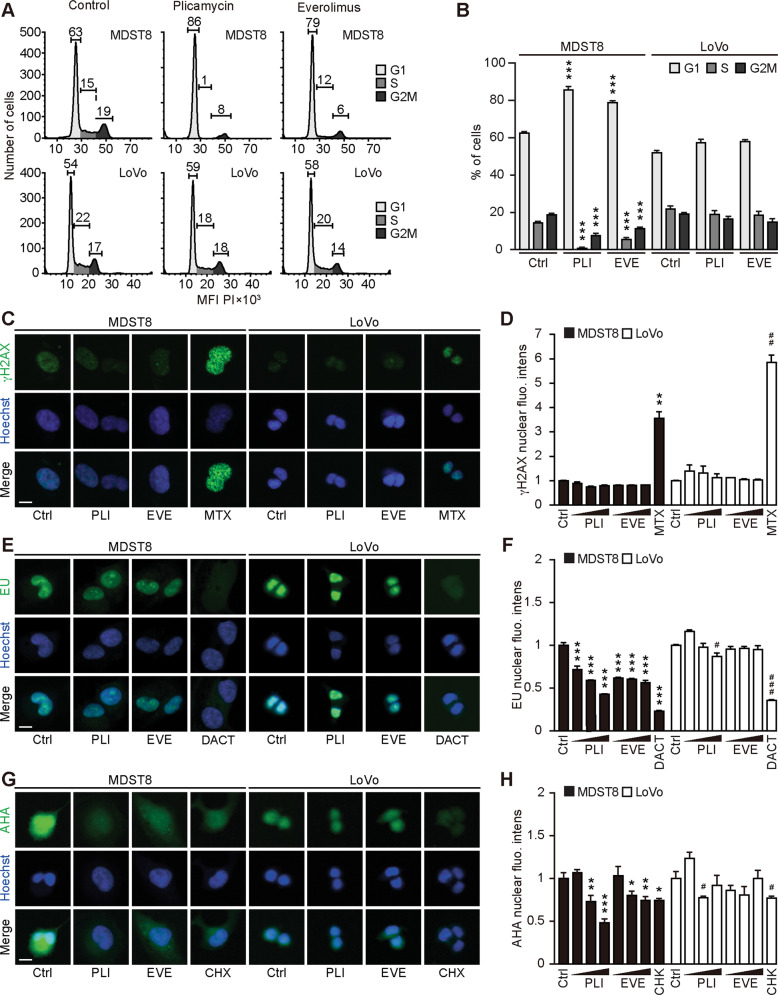
Cellular stress response to everolimus and plicamycin. **A**, **B** Alterations in the cell cycle progression in response to plicamycin (PLI) or everolimus (EVE) were studied by flow cytometry. Human colon cancer MDST8 and LoVo cells were treated with 50 nM PLI or 100 nM EVE for 48 h, then fixed and stained with FxCycle™ PI/RNase, followed by flow cytometric assessment. Representative cell cycle histograms of MDST8 and LoVo cells are shown in (**A**) and the percentage of cells in each cell cycle phase are depicted as a bar chart in (**B**). Error bars indicate SEM. Asterisks refer to significant effects for treatments versus control (paired Student’s *t* test, *P < 0.05, **P < 0.01, ***P < 0.001). **C**–**H** MDST8 and LoVo cells were pre‐treated with EVE at 0.01, 0.1, and 1 μM, or with PLI at 12.5, 25, and 50 nM for 24 h; with mitoxantrone (MTX) at 1 μM for 16 h; with dactinomycin (DACT) at 2 μM, or cycloheximide (CHX) at 50 μM for 6 h followed by fixation and permeabilization. Then, cells were incubated with a rabbit anti‐phospho-histone H2A.X (γH2A.X) antibody and stained with an anti‐rabbit Alexa Fluor‐488‐coupled secondary antibody. The formation of nuclear γH2A.X^+^ foci is shown in (**C**) and the average nuclear intensity of the γH2A.X signal was quantified (**D**). Cells were pre‐treated with the aforementioned compounds in a complete medium and followed by an additional hour of treatment in the presence of 100 mM 5‐ethynyl uridine (EU). After fixation, cells were permeabilized, and EU was stained with an Alexa Fluor‐488‐coupled azide. Representative images are shown for each treatment (**E**). The EU intensity in the nucleus of each condition was ranked between the untreated control (control, Ctrl, 0% transcription inhibition) and the control that was not incubated with EU (corresponding to 100% transcription inhibition) (**F**). Cells were pre‐treated with the aforementioned compounds in complete medium followed by washout and treatment pursued in the methionine‐free medium for 30 min. Afterward, the treatments were continued in methionine‐free medium supplemented with 50 μM L‐azidohomoalanine (AHA) for 1 h and AHA incorporation was detected after fixation, permeabilization, and blocking by the addition of an Alexa Fluor‐488‐coupled azide. Then, images were acquired (**G**), and AHA intensity in the cells was ranked between the untreated control (Ctrl, 0% translation inhibition) and control without AHA (corresponding to 100% translation inhibition) (**H**). Data information: representative images of EVE 1 μM, PLI 50 nM and MTX 1 μM are shown (**C**); EVE 0.1 μM, PLI 25 nM, and DACT 2 μM are shown (**E**); EVE 1 μM, PLI 50 nM, and CHX 50 μM are shown (**G**). Scale bars represent 20 μm. One representative experiment among three is shown as mean ± SD, and *P*‐values indicating differences to controls were calculated with Student’s *t* test: *P < 0.05, **P < 0.01, ***P < 0.001 versus untreated MDST8 control; ^#^*P* < 0.05, ^#^^#^*P* < 0.01, ^#^^#^^#^*P* < 0.001 versus untreated LoVo control (**D**, **F**, **H**).

**Fig. 5:**
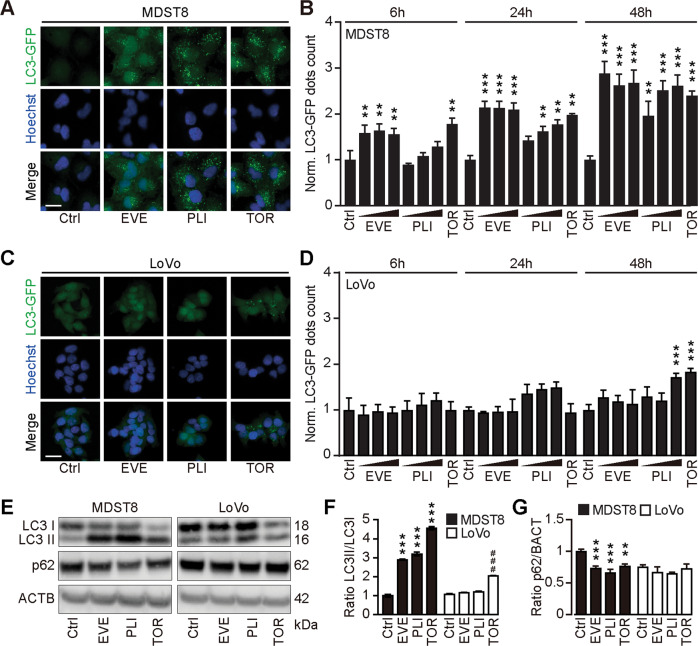
Everolimus induces autophagy in MDST8. **A** MDST8 and **C** LoVo cells stably expressing GFP-LC3 were treated with plicamycin (PLI; 25, 50, and 100 nM), everolimus (EVE; 10, 100 nM and 1 µM) or torin1 (TOR; 0.1 μM) for 6 h 24 h and 48 h. After fixation and nuclear staining with Hoechst 33342, the images were acquired by confocal microscopy. Representative images are depicted for each cell line. The scale bar equals 20 μm. **B**, **D** GFP- LC3 dots area were quantified. For each assessed parameter and cell line, data were normalized to the untreated control. Data represent means ± SD. Each condition was compared to the untreated control by means of a paired Student’s *t* test (**P < 0.01, ***P < 0.001). **E**–**G** Human colon cancer MDST8 or LoVo cells were treated with EVE (0.1 μM) or PLI (50 nM) for 72 h. TOR (300 nM) was used for 6 h as a prototypical autophagy inducer. SDS–PAGE and immunoblot were performed, band intensities of LC3-I, LC3-II, p62, and β-actin (ACTB) were assessed, and the ratio LC3-II/ LC3-I (**F**) and p62/ACTB (**G**) were calculated. Data are means ± SD of three independent experiments (**P < 0.01, ***P < 0.001 versus untreated MDST8 control; ^###^*P* < 0.001 versus untreated LoVo control; Tukey’s multiple comparisons test).

We continued the comparative analysis of clinically approved EVE and PLI on LoVo and MDST8 cells to characterize specific vulnerabilities of the latter cell line. Annexin V-AF647/DAPI staining revealed that MDST8 cells were selectively killed by plicamycin while presenting both early apoptotic (Annexin V-AF647^+^DAPI^−^) and necrotic (Annexin V-AF647^+^DAPI^+^) events. In contrast, MDST8 cells were resistant against the anticancer agents oxaliplatin (OXA) and sunitinib (SUN) in conditions in which a sizeable fraction of LoVo cells died (Fig. 2A–C). The differential PLI sensitivity (and SUN resistance) of CMS4 cells over CMS1 cells was confirmed for another pair of human colorectal cancer cell lines, namely, Colo320HSR and HCT116, which represent the CMS4 and CMS1 subtypes, respectively (Supplementary Fig. S3). Moreover, PLI (and to less degree EVE) induced a higher level of caspase-3 activation (measured with a fluorogenic substrate) in MDST8 than in LoVo cells (Fig. 2D, E), and PLI (and to less degree EVE) caused the release of cytochrome C from mitochondria (measured by an immunofluorescence assay that assesses the reduction of the staining intensity) more efficiently in MDST8 than in LoVo cells (Fig. 3). Moreover, MDST8 but not LoVo cells manifested an elongation of mitochondria stained with MitoTracker, as well as a reduction of MitoTracker staining (Supplementary Fig. S4). Other cellular assays confirmed the selective susceptibility of MDST8 cells to EVE and PLI as compared to LoVo cells. Thus, both EVE and PLI caused an accumulation of cells in the G0/G1 phase of the cell cycle (measured by propidium iodide staining of ethanol-permeabilized, RNase-treated cells, and cytofluorometry) with a concomitant reduction of cells in the S and G2/M phase in MDST8 but not in LoVo cells (Fig. 4A, B). Although neither EVE nor PLI induced DNA damage assessed by immunofluorescence detection of nuclear γ-histone 2 A.X foci (Fig. 4C, D), both agents caused a reduction in DNA-to-RNA transcription and RNA-to-protein translation in MDST8 but not in LoVo cells, as measured by quantifying the cellular incorporation of the RNA precursor ethacrynic uridine (EU) and the protein precursor L-azidohomoalanine (AHA), respectively (Fig. 4E–H). Finally, the autophagy-association redistribution of microtubule-associated proteins 1A/1B light chain 3B (hereafter referred to as LC3) fused to GFP (GFP-LC3), the lipidation of LC3 causing an increase in its electrophoretic mobility (annotated as LC3-II), and the decrease in the autophagic substrate sequestosome-1 (SQSTM1, best known as p62) were observed in MDST8 but not in LoVo cells cultured with EVE or PLI (Fig. 5). Altogether, these results demonstrate that MDST8 cells are sensitive to the induction of cytostatic cell stress and cell death by EVE and PLI, respectively.

### In vivo treatment of MDST8 tumors with everolimus and plicamycin

**Fig. 6:**
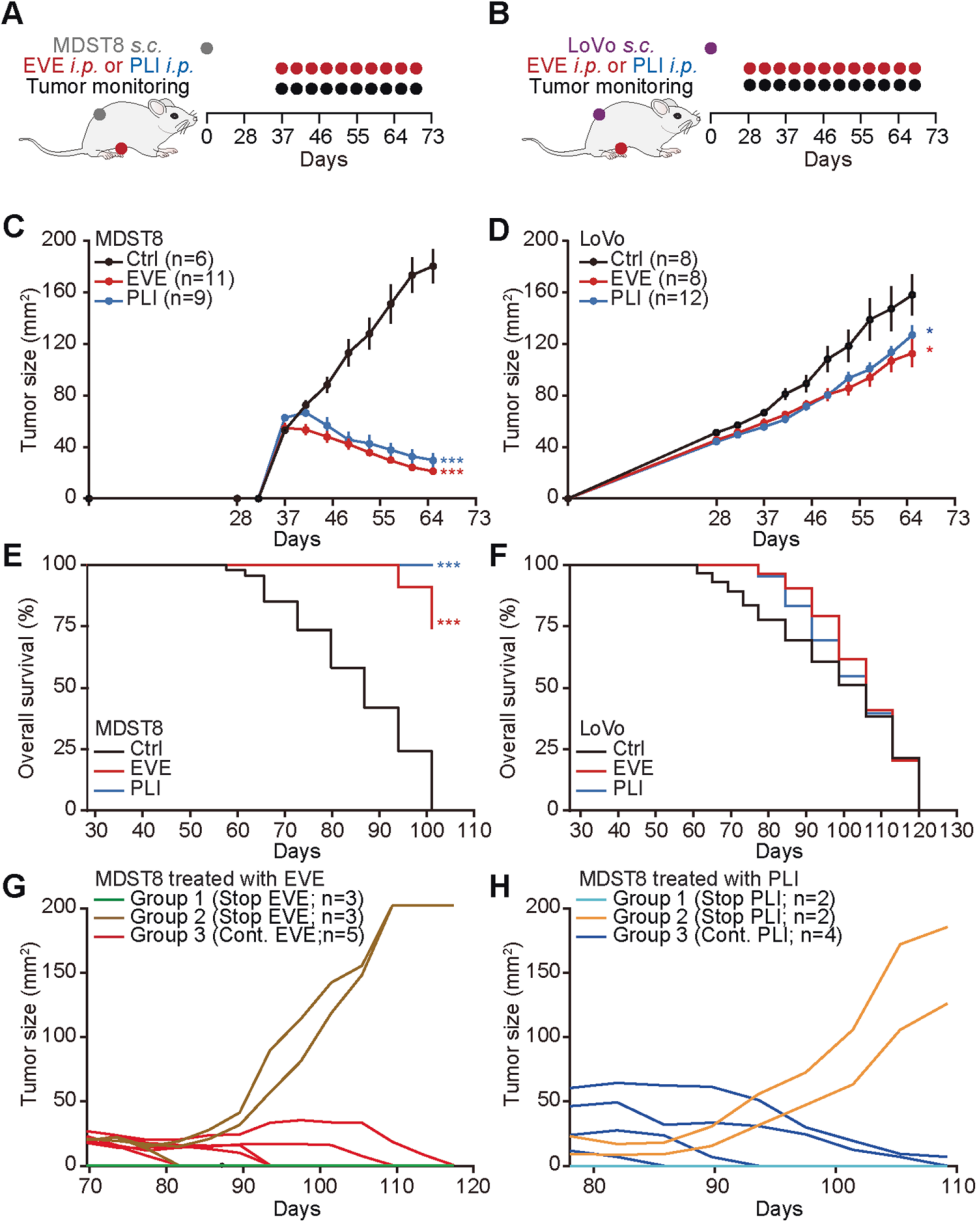
Everolimus and plicamycin exhibit anticancer effects against CMS4 tumors. **A**, **B** Schematic overview of the treatment schedule of LoVo or MDST8 tumors with everolimus (EVE) and plicamycin (PLI) in vivo. **C**–**H** Five million human colon cancer MDST8 or LoVo cells were injected subcutaneously (s.c.) into the flank of athymic immunodeficient *nu/nu* mice. When tumors became palpable, mice received a systemic intraperitoneal injection of EVE or PLI. *n* ≥ 6 mice per group. Results (means ± SD tumor growth curves) are plotted (*P < 0.05, ***P < 0.001). Overall survival is depicted, and *P* values (***P < 0.001) were calculated with a Log‐rank test (**E**, **F**). After EVE/PLI treatment, mice bearing MDST8 tumors were divided into three different groups, and tumor growth was monitored upon continuation or discontinuation of the treatment as indicated (**G**, **H**).

As a final proof that MDST8 tumors can be treated with the drugs identified in this study, we inoculated mice with MDST8 or, as a control, LoVo cells. Once palpable tumors had been established, the mice received systemic injections of either EVE or PLI on a biweekly basis (Fig. 6A, B). While MDST8 tumors reduced their volume in response to EVE and PLI, LoVo tumors continued their progression (Fig. 6C, D and Supplementary Fig. S5A, B). This drug effect was not accompanied by any manifest signs of toxicity (and in particular weight loss, Supplementary Fig. S5C, D) and caused a significant extension of lifespan in mice carrying MDST8 but not LoVo tumors (Fig. 6E, F). In a limited number of cases, we stopped the treatment of MDST8-bearing mice at day 65 post-inoculation. For those mice that lacked palpable tumor masses after EVE or PLI treatment, discontinuation of the drugs did not result in recurrence of the tumors, suggesting that these animals had been definitively cured from their cancers. In contrast, when macroscopic tumors had not been fully eliminated, discontinuation of EVE or PLI resulted in regrowth of most cancers, contrasting with the continuous shrinkage of the majority of tumors that underwent further therapy (Fig. 6G, H). These results suggest that tumors usually remained sensitive to EVE and PLI throughout the treatment phase, for up to 3 months (from day 37 to day 117). Altogether, these results demonstrate that MDST8 tumors can be held in check by continuous, nontoxic administration of EVE and PLI.

## Discussion

This work demonstrates that two mechanistically unrelated drugs, everolimus (EVE, an inhibitor of mTORC1) and plicamycin (PLI, a DNA-binding agent that inhibits RNA synthesis) efficiently target the CMS4 cell line MDST8, both in vitro and in vivo. It will be interesting to determine the molecular mechanisms explaining why MDST8 cells are selectively susceptible to these agents. Moreover, it will be important to evaluate the potential clinical utility of these agents for the treatment of CMS4 colorectal cancers.

Everolimus is FDA approved for a series of indications including hormone receptor-positive, HER2-negative advanced breast cancer (in combination with aromatase inhibitors), neuroendocrine tumors (NET) of gastrointestinal (GI) or lung origin, advanced renal carcinoma, renal angiomyolipoma associated with tuberous sclerosis complex (TSC), subependymal giant cell astrocytoma (SEGA) associated with TSC [23]. Clinical trials on colorectal cancer patients largely failed when EVE was used as a single agent [24, 25] or combined with the anti-VEGF-A antibody bevacizumab [26] or the insulin receptor/insulin-like growth factor R receptor inhibitor linsitinib [27] for the treatment of refractory metastatic colorectal cancer. However, stable disease was induced in 50% of patients with refractory metastatic colorectal cancer when EVE was combined with tivozanib (an oral VEGF receptor-1, -2, -3 inhibitor) [28], and a 60% response rate was reported when EVE was combined with the chemotherapeutic agent irinotecan and the anti-EGFR antibody panitumamab [29]. Currently, there is one clinical trial (NCT02890069) that recruits colorectal cancer patients to combine EVE with the PD-1-blocking antibody PDR001. It may be interesting to apply the CMS classification to these trials and to re-evaluate the possibility that patients bearing cancers falling into the CMS4 category obtain clinical benefit from treatment with EVE alone or in combination with other agents.

Plicamycin (which is often referred to as “mithramycin A”) has been clinically evaluated for the treatment of Ewing sarcoma (NCT01610570), as well as for the treatment of lung, esophagus, and other thoracic cancers (NCT01624090). A Phase I/II that is currently recruiting patients with primary thoracic malignancies or extrathoracic neoplasias with pleuropulmonary metastases evaluates the effects of continuous intravenous infusion of mithramycin (NCT02859415). However, PLI has not been evaluated in the context of colorectal cancer, apart from one phase II study reporting a major regression of one rectal adenocarcinoma in response to this agent [30]. Of note, this inhibitor of DNA-to-RNA transcription has been reported to target colorectal cancer stem cells [31], perhaps due to the inhibition of the transcription factor Sp1 [32]. Interestingly, it appears that inhibition of transcription by plicamycin is well detectable in the susceptible CMS4 cell line MDST8 but not in the resistant CMS1 cell line LoVo.

It will be important to evaluate whether the mechanism that we explored here comes into action in vivo and whether patients with CMS4 colorectal cancer might benefit from PLI, alone or in combination with EVE. Indeed, in the xenograft models, both PLI and EVE exhibit satisfactory preclinical activity against CMS4 cancers. Future clinical trials might establish whether these two drugs can be advantageously combined to achieve efficient tumor shrinkage without major side effects.

## Materials and methods

### Cell lines

Human colon Colo320HSR, HCT116, LoVo, and MDST8 cells were purchased from the American Type Cancer Collection (ATCC). MDST8 and LoVo wild-type cells were transduced with LentiBrite™ H2B-RFP and H2B-GFP lentiviral particles (Merck Millipore, Burlington, MA, USA), respectively, following the manufacturer’s instructions, to obtain MDST8 H2B-RFP and LoVo H2B-GFP. In addition, both MDST8 and LoVo wild-type cells were transduced with LentiBrite™ GFP-LC3 lentiviral particles (Merck Millipore, Burlington, MA, USA), to obtain MDST8 GFP-LC3 and LoVo GFP-LC3 cells, as described [33–35].

### Cell culture

MDST8 and MDST8 GFP-LC3 cells were cultured in Dulbecco’s Modified Eagle medium with high glucose (Thermo Fisher Scientific, Carlsbad, CA, USA) while the medium of LoVo and LoVo GFP-LC3 was Ham’s F-12K (Kaighn’s) (Thermo Fisher Scientific). Both media were supplemented with 10% fetal bovine serum (Gibco^®^ Thermo Fisher Scientific), 10 U/mL penicillin sodium, and 10 U/mL streptomycin sulfate (Thermo Fisher Scientific), and cells were kept in a humidified incubator with 5% CO_2_ at 37 °C. Cell culture plastic was purchased from Corning (Corning, NY, USA) and Greiner Bio-One (Kremsmünster, Austria).

### Compounds and reagents

A custom-arrayed anticancer library was used [36]. Oxaliplatin came from Accord Healthcare (Ahmedabad, India). Sunitinib (PZ0012), crizotinib (PZ0191), 5-fluorouracil (F6627), everolimus (SML2282), rapamycin (R8781), plicamycin (M6891), staurosporine (S5921), thapsigargin (T9033) methotrexate (M7824), and DMSO were purchased from Sigma-Aldrich. The MAD2 inhibitor M2I-1 (312271-03-7) was from Cayman. Everolimus (HY-10218) and plicamycin (HY-A0122) for in vivo experimentation were purchased from MedChemExpress. Hoechst 33342 (H3570) and Lipofectamine^®^ 2000 were purchased from Thermo Fisher Scientific. Propidium iodide (P4864), formaldehyde (F8775), and Triton X-100 (T8787) were purchased from Sigma-Aldrich (St. Louis, MO, USA).

### Flow cytometric analysis

For high-throughput screening, cancer cells were seeded in 96-well plates (1 × 10^4^ cells/well) in 100 μL cell culture medium and let adapt for 24 h before treatment. Then cells were treated with the 71 chemicals of the anticancer library at 0.1 μM, 1 μM, or 10 μM final concentration for 48 h or 72 h. Then cells were collected in 96-well V-shape plates (Greiner Bio-One, Frickenhausen, Germany), washed with PBS, and then the cell pellets were resuspended in 100 μL Annexin V Binding Buffer (422201, Biolegend) containing 0.2 μL Annexin V (640919, Biolegend) and 0.1 μL DAPI. Samples were then incubated in the dark for 15 min. After that, the plates were immediately subjected to flow cytometry acquisition using a high-throughput sampler mounted on a BD LSRFortessa flow cytometer (Beckton Dickinson, Franklin Lakes, NY, USA). Data were further processed with the FlowJo software (LLC, Ashland, OR, USA) to assess the percentage of Annexin V^+^ and DAPI^+^ dying and dead cells, respectively [37]. Then the data were imported into the free available software R (https://www.r-project.org) and integrated with the heatmap packages from the Bioconductor repository (https://bioconductor.org/) to graphically depict data as a heatmap.

### Assessment of caspase activity

Cells were seeded in 12-wells plates (5 × 10^4^ cells/well). The next day, cells were treated with 0.1 μM everolimus, 100 nM plicamycin, or 0.1 μM staurosporine for 48 h. After that, cells were collected and fixed with intracellular (IC) Fixation Buffer (00-8222-49, Invitrogen) and permeabilized with Permeabilization Buffer (00-8333-56, Invitrogen) and finally stained with a rabbit anti-human/mouse caspase-3 Alexa Fluor^®^ 488-conjugated monoclonal antibody (IC835G, Invitrogen) for flow cytometric analysis. The mean fluorescence intensity was analyzed with the FlowJo software.

### Cell cycle analysis

Cells were seeded in 12-wells plates (5 × 10^4^ cells/well) and let adapt overnight. The next day, cells were treated with 0.1 μM everolimus, 50 nM plicamycin, or 5 μM sunitinib for 48 h. After the treatment, the supernatant was discarded and the cells were collected and transferred into flow cytometry tubes. Cells were agitated and fixed in cold 70% ethanol for 2 min and kept in the dark at 4 °C overnight. Then the cells were washed three times with PBS and resuspended in 500 µL FxCycle™ PI/RNase staining solution (F10797, Thermo Fisher). The samples were incubated for 15–30 min at room temperature, protected from light, and finally analyzed on a Cytoflex (Beckman Coulter) flow cytometer. Data analysis was performed with the FlowJo software.

### High-throughput screening

Wild-type cells were seeded in 384-well black imaging plates (Greiner Bio-One) at a density of 1.5 × 10^3^ cells/well and let adhere for 24 h. The next day, cells were treated with drugs of an anticancer compound library in 0.1 µM concentration for 72 h. For viability assessment, cells were fixed with 3.7% formaldehyde containing 1 μg/mL mL Hoechst 33342 for 1 h at room temperature. The fixative was exchanged to PBS and viability was assessed by automated microscopy.

### Automated fluorescence microscopy

MDST8 GFP-LC3 or LoVo GFP-LC3 cells were seeded in 96-well black imaging plates at a density of 1.5 × 10^3^ cells/well. The next day, cells were treated with everolimus (10, 100 nM, and 1 µM), plicamycin (25, 50, and 100 nM), or torin (0.3 μM), and incubated for 6, 24 or 48 h. After that, cells were stained with MitoTracker™ Orange (M7510, Thermo Fisher) [38] and then fixed with 3.7% formaldehyde containing Hoechst 33342. Automated fluorescence microscopy was conducted by means of a robot-assisted Molecular Devices IXM XL BioImager and a Molecular Devices IXM-C (Molecular Devices, Sunnyvale, CA, USA) equipped with either a SpectraX or an Aura II light source (Lumencor, Beaverton, OR, USA), adequate excitation and emission filters (Semrock, Rochester, NY, USA) and a 16-bit monochromes sCMOS PCO.edge 5.5 camera (PCO Kelheim, Germany) or an Andor Zyla camera (Belfast, Northern Ireland) and a ×20 PlanAPO objective (Nikon, Tokyo, Japan) were used to acquire a minimum of four view fields per well, followed by automated image processing with the custom module editor within the MetaXpress software (Molecular Devices) and/or R employing the EBImage and RBioFormats packages. Image segmentation was performed using the MetaXpress software (Molecular Devices). Following the exclusion of cellular debris and dead cells from the dataset, parameters of interest were normalized, statistically evaluated, and graphically depicted with R software [39]. Cytoplasmic ROIs were used for the quantification of cytochrome *c* intensity. To quantify GFP-LC3 aggregation, a segmentation mask of high-intensity dots was generated in the cytoplasm of cells.

### Monitoring mitochondrial cytochrome *c* release

Wild-type cells were plated onto coverslips previously coated with 10 µg/mL poly-l-lysine in a 12-well plate. The next day, cells were treated with 0.1 μM everolimus, 50 nM plicamycin, or 0.1 μM staurosporine for 24 h or 48 h. After the treatment, cells were stained with MitoTracker, fixed with 3.7% formaldehyde, as described previously, and permeabilized with 0.2% Triton X-100 for 10 min. Then the cells were incubated with Alexa Fluor^®^ 647 coupled anti-cytochrome *c* antibody (612310, Biolegend) overnight at 4 °C in the dark. Finally, cells were washed with PBS and mounted with Fluoromount-G™ mounting medium (00-4958-02, Thermo Fisher). Fluorescence confocal microscopy was carried out using a Leica TCS SP8 Confocal Microscope with a ×63 oil immersion objective (Leica Microsystems, Wetzlar, Germany). Images were acquired from randomly selected fields of cells. Subsequently, the percentage of each subtype was evaluated for each treatment and a minimum of 30 cells were considered for the analysis. Image analysis was performed with the LAS X software (Leica) and R.

### Clonogenic assay

MDST8 and LoVo cells were seeded in six-well plates at 1 × 10^3^ cells per well. After 24 h, cells were treated with 10 nM everolimus, 10 nM plicamycin, or 2 μM sunitinib for 3 weeks (MDST8) or 4 weeks (LoVo). After that, the supernatant was discarded and the cells were incubated with 500 µL of crystal violet (Sigma) for 10 min. Then, cells were washed with deionized water, images were acquired and the area of each colony was quantified through Fiji’s ColonyArea plugin, as described [40].

### Quantitative RT-PCR

Total RNA extraction of cultured cells was performed with the GeneJET RNA Purification Kit (Life Technologies). In total, 2.5 μg RNA was then reverse transcribed into cDNA with the Maxima First Strand cDNA Synthesis Kit (Life Technologies). The expression of the genes of interest (Table 1) was analyzed by means of SYBR^®^ green-based quantitative PCR using the Power SYBRTM Green PCR Master Mix in a StepOnePlus Real-Time PCR System (Applied Biosystems, Forster City, CA, USA). qRT-PCR data were normalized to the expression levels of the housekeeping gene hypoxanthine phosphoribosyltransferase 1 (HPRT1) and data were depicted as a Volcano plot employing R.

**Table 1:** RT-qPCR primers [45].

Human gene	Forward sequence	Reverse sequence
HPRT1	CCTGGCGTCGTGATTAGTGA	CGAGCAAGACGTTCAGTCCT
CDH2	ACAGTGGCCACCTACAAAGG	CCGAGATGGGGTTGATAATG
SLUG	GGTCAAGAAGCATTTCAACG	CACAGTGATGGGGCTGTATG
VIM	CCCTCACCTGTGAAGTGGAT	TCCAGCAGCTTCCTGTAGGT
MMP2	TCTCCTGACATTGACCTTGGC	CAAGGTGCTGGCTGAGTAGATC
MMP9	TTGACAGCGGACAAGAAGTGG	GCCATTCACGTCGTCCTTAT
MMP13	TCCCAGGAATTGGTGATAAAGTAGA	CTGGCATGACGCGAACAATA
CYP1B1	CACTGCCAACACCTCTGTCTT	CAAGGAGCTCCATGGACTCT
GAS1	AAAGTCTTCAACGGGCTGCGCT	TCCTTGACCGACTCGCAGATGG
HTR2B	TGTCCTTGGCGGTGGCTGAT	TGGCACAGAGATGCATGATGGA
RGS4	AACACAATTCTTCCCACAACAA	CTGCCAGCCCACATTCA
FRMD6	AAGGACTGCCACCTCTTTGG	AGTTCCCAAGATCAGCCTGC
INHBA	GCAGTCTGAAGACCACCCTC	ATGATCCAGTCATTCCAGCC
CDX2	TTCACTACAGTCGCTACATCACC	TTGTTGATTTTCCTCTCCTTTGC
ZEB1	GCACAAGAAGAGCCACAAGTA	GCAAGACAAGTTCAAGGGTTC
SP1	TGGCAGCAGTACCAATGGC	CCAGGTAGTCCTGTCAGAACTT
HSP70	AGCTGGAGCAGGTGTGTAAC	CAGCAATCTTGGAAAGGCCC
MAD2L1	TTCTCATTCGGCATCAAC	TCCAGGACCTCACCACTT
AIF1	GTTCCAGCGATGGCATGTTC	ACGCGGCCTTTTTCTGTTTC
HOPX	GCTCATTTTCCTGGGCTGTTA	GGATTTCCACCTGGTCCTCTG

Primers utilized for the detection of CMS-related mRNA expression profiles.

### Protein immunoblots

Protein was extracted with RIPA lysis and extraction Buffer (89900; Thermo Scientific) in the presence of phosphatase and protease inhibitors (A32961; Thermo Scientific) followed by sonication. Then, protein content was measured by a DC™ Protein Assay Kit II (5000112; Bio-Rad) following the manufacturer’s protocol. Protein was denatured at 100 °C, and 30 μg of proteins and 10 μL PAGE Ruler prestained protein ladder (26616; Thermo Scientific) were separated by polyacrylamide gel electrophoresis (PAGE) using 4–12% Bis-Tris Novex™ NuPAGE™ protein gels (NP0336PK2; Invitrogen) in Novex™ NuPAGE™ MES SDS migration buffer (1×) (NP000202; Invitrogen). Afterward, proteins were transferred to EtOH‐activated PVDF membranes (88518; Thermo Scientific) in transfer buffer (25 mM Tris; 190 mM glycine; 10% ethanol in H_2_O) at 200 mA and 120 V for 1.5 h. Membranes were washed in Tris‐buffered saline with Tween-20 buffer (TBST; 20 mM Tris, pH 7.5; 150 mM NaCl; 0.1% Tween-20 in H_2_O) and then blocked with 5% skim milk in TBST for 1 h. Membranes were exposed to primary antibody (anti-LC3B antibody; ab192890; Abcam) at 1:2000; p62/SQSTM1 monoclonal antibody (H00008878-M01, Abnova) at 1:1000) diluted in 5% BSA in TBST overnight at 4 °C. Next, membranes were washed three times with TBST and then were incubated with 1:25000 appropriate horseradish peroxidase (HRP)‐coupled secondary antibody (goat anti-rabbit IgG (H + L) (4050-05, SouthernBiotech); goat anti-mouse IgG (H + L) (1031-05, SouthernBiotech)) for 1 h at room temperature. Proteins were revealed with Amersham ECL Prime Western Blotting Detection Reagent (RPN2232; GE Healthcare Life Sciences). Anti-beta actin antibody (ab49900; Abcam) at 1:50,000 was used to verify equal loading.

### Evaluation of DNA damage by quantification of phospho-histone H2A.X

Two thousand cells per well were cultured in 384‐well μClear imaging plates. The next day, cells were treated for 24 h. Following, cells were fixed with 3.7% formaldehyde supplemented with 1 μg/mL Hoechst 33342 for 1 h, permeabilized with 0.5% Triton X‐100 for 15 min and blocked with 3% BSA for 1 h. Cells were further incubated with 1:1000 rabbit antibody specific for phospho-histone H2A.X (**γ**H2A.X) overnight at 4 °C. After several PBS washing steps, 1:2000 anti‐rabbit Alexa Fluor‐488‐coupled antibodies were added. Following several PBS washing steps, the DAPI and GFP signals were acquired with a confocal microscope IXM‐C (Molecular Devices) and quantified as described before [41, 42].

### Evaluation of RNA transcription by EU incorporation

Transcription was analyzed by measuring the incorporation of Click‐iT chemistry‐detectable 5‐ethynyl uridine (EU) (C10327; Invitrogen) as described before [43]. In short, 2 × 10^3^ cells per well were seeded in 384‐well μClear imaging plates. The next day, cells were pre‐treated for 24 h and washed and treatment was pursued in the presence of 1 mM 5‐ethynyl uridine (EU) for 1 h. Following, the cells were fixed with 3.7% formaldehyde supplemented with 1 μg/mL Hoechst 33342 for 1 h and permeabilized with 0.5% Triton X‐100 for 15 min. Alexa Fluor‐488‐coupled azide was then added for 1 h. The intensity of the GFP signal (EU) in the nucleus was measured by microscopy, and the inhibition of transcription was calculated as a fold change in fluorescence intensity as compared to controls.

### Protein translation study by AHA incorporation

Translation was measured by assessing the incorporation of L‐azidohomoalanine (AHA) (C10289; Invitrogen), a labeled form of methionine by Click‐iT chemistry as described [44]. In short, 2 × 10^3^ cells per well were seeded in 384‐well μClear imaging plates. The next day, cells were treated for 24 h. After several PBS washing steps, the cells were incubated 30 min in the presence of methionine‐free medium. They were further treated for 1 h in methionine‐free medium in the presence of 50 μM AHA. Afterward, the cells were fixed with 3.7% formaldehyde supplemented with 1 μg/mL Hoechst 33342 for 1 h, permeabilized with 0.5% Triton X‐100 for 15 min, and blocked with 3% BSA for 1 h. Then, Alexa Fluor‐488‐coupled azide was added for 1 h and AHA incorporation was measured by microscopy as a fold change in GFP fluorescence intensity.

### In vivo tumor treatment

Established tumors were assessed for their response to everolimus- and plicamycin‐based chemotherapy. To this aim, colon cancers were established subcutaneously (s.c.) in athymic *nu/nu* mice by injection of 5 × 10^6^ MDST8 or LoVo cells. When tumors became palpable, 200 μL of the chemotherapeutics (everolismus diluted in 90% corn oil, 4 mg/kg; plicamycin diluted in 40% PEG300, 5% Tween-80 and 45% saline, 1.5 mg/kg) or the diluent alone were injected intraperitoneally (i.p.) and tumor growth was monitored for the following weeks [5].

### Experimental animals

In vivo experimentation. Seven- to eight-week-old female wild-type *nu/nu* mice were purchased from Envigo France (Gannat, France) and were kept at the Gustave Roussy Campus Cancer in a specific pathogen-free and environmental temperature-controlled animal facility with 12 h day, 12 h night cycles, and received food and water ad libitum. Animal experiments were conducted in compliance with the EU Directive 63/2010 and were approved by the Ethical Committee of the Gustave Roussy Campus Cancer (CEEA IRCIV/IGR no. 26, registered at the French Ministry of Research).

### Statistical analysis

Unless otherwise mentioned, data are reported as means ± SD of triplicate determinations, and experiments were repeated at least three times yielding similar results. Statistical significance was assessed by Welch’s and Student’s *t* test. TumGrowth and GraphPad were used to analyze in vivo data raised in murine models [5]. TumGrowth is available at https://github.com/kroemerlab. *P* values of 0.05 or less were considered to denote significance (*P < 0.05; **P < 0.01; ***P < 0.001; ns, not significant).

## Supplementary information

Supplemental Figure Legends

Figure S1

Figure S2

Figure S3

Figure S4

Figure S5

## Data Availability

Data are available from the corresponding authors upon reasonable request.

## References

[1] Markowitz SD, Bertagnolli MM (2009). Molecular origins of cancer: molecular basis of colorectal cancer. N. Engl J Med.

[2] Dekker E, Tanis PJ, Vleugels JLA, Kasi PM, Wallace MB (2019). Colorectal cancer. Lancet.

[3] Bos JL (1989). ras oncogenes in human cancer: a review. Cancer Res.

[4] Galon J, Costes A, Sanchez-Cabo F, Kirilovsky A, Mlecnik B, Lagorce-Pages C (2006). Type, density, and location of immune cells within human colorectal tumors predict clinical outcome. Science.

[5] Enot DP, Vacchelli E, Jacquelot N, Zitvogel L, Kroemer G (2018). TumGrowth: an open-access web tool for the statistical analysis of tumor growth curves. Oncoimmunology.

[6] Loeb LA (1994). Microsatellite instability: marker of a mutator phenotype in cancer. Cancer Res.

[7] Lievre A, Bachet JB, Le Corre D, Boige V, Landi B, Emile JF (2006). KRAS mutation status is predictive of response to cetuximab therapy in colorectal cancer. Cancer Res.

[8] Lievre A, Bachet JB, Boige V, Cayre A, Le Corre D, Buc E (2008). KRAS mutations as an independent prognostic factor in patients with advanced colorectal cancer treated with cetuximab. J Clin Oncol.

[9] Anitei MG, Zeitoun G, Mlecnik B, Marliot F, Haicheur N, Todosi AM (2014). Prognostic and predictive values of the immunoscore in patients with rectal cancer. Clin Cancer Res.

[10] El Sissy C, Kirilovsky A, Zeitoun G, Marliot F, Haicheur N, Lagorce-Pages C, et al. Therapeutic implications of the immunoscore in patients with colorectal cancer. Cancers. 2021;13:1281. 10.3390/cancers13061281 PMC8001764 33805758

[11] Kroemer G, Galluzzi L, Zitvogel L, Fridman WH (2015). Colorectal cancer: the first neoplasia found to be under immunosurveillance and the last one to respond to immunotherapy?. Oncoimmunology.

[12] Bilgin B, Sendur MA, Bulent Akinci M, Sener Dede D, Yalcin B (2017). Targeting the PD-1 pathway: a new hope for gastrointestinal cancers. Curr Med Res Opin.

[13] Thomas J, Leal A, Overman MJ (2020). Clinical development of immunotherapy for deficient mismatch repair colorectal cancer. Clin Colorectal Cancer.

[14] Guinney J, Dienstmann R, Wang X, de Reynies A, Schlicker A, Soneson C (2015). The consensus molecular subtypes of colorectal cancer. Nat Med.

[15] Becht E, de Reynies A, Giraldo NA, Pilati C, Buttard B, Lacroix L (2016). Immune and stromal classification of colorectal cancer is associated with molecular subtypes and relevant for precision immunotherapy. Clin Cancer Res.

[16] Thanki K, Nicholls ME, Gajjar A, Senagore AJ, Qiu S, Szabo C (2017). Consensus molecular subtypes of colorectal cancer and their clinical implications. Int Biol Biomed J.

[17] Marisa L, Svrcek M, Collura A, Becht E, Cervera P, Wanherdrick K, et al. The balance between cytotoxic T-cell lymphocytes and immune checkpoint expression in the prognosis of colon tumors. J Natl Cancer Inst. 2018;110:68–77. 10.1093/jnci/djx136 28922790

[18] Sveen A, Bruun J, Eide PW, Eilertsen IA, Ramirez L, Murumagi A (2018). Colorectal cancer consensus molecular subtypes translated to preclinical models uncover potentially targetable cancer cell dependencies. Clin Cancer Res.

[19] Linnekamp JF, Hooff SRV, Prasetyanti PR, Kandimalla R, Buikhuisen JY, Fessler E (2018). Consensus molecular subtypes of colorectal cancer are recapitulated in in vitro and in vivo models. Cell Death Differ.

[20] Okita A, Takahashi S, Ouchi K, Inoue M, Watanabe M, Endo M (2018). Consensus molecular subtypes classification of colorectal cancer as a predictive factor for chemotherapeutic efficacy against metastatic colorectal cancer. Oncotarget.

[21] Mooi JK, Wirapati P, Asher R, Lee CK, Savas P, Price TJ (2018). The prognostic impact of consensus molecular subtypes (CMS) and its predictive effects for bevacizumab benefit in metastatic colorectal cancer: molecular analysis of the AGITG MAX clinical trial. Ann Oncol.

[22] Bramsen JB, Rasmussen MH, Ongen H, Mattesen TB, Orntoft MW, Arnadottir SS (2017). Molecular-subtype-specific biomarkers improve prediction of prognosis in colorectal cancer. Cell Rep..

[23] Hasskarl J (2018). Everolimus. Recent Results Cancer Res.

[24] Altomare I, Hurwitz H (2013). Everolimus in colorectal cancer. Expert Opin Pharmacother.

[25] Ng K, Tabernero J, Hwang J, Bajetta E, Sharma S, Del Prete SA (2013). Phase II study of everolimus in patients with metastatic colorectal adenocarcinoma previously treated with bevacizumab-, fluoropyrimidine-, oxaliplatin-, and irinotecan-based regimens. Clin Cancer Res.

[26] Altomare I, Bendell JC, Bullock KE, Uronis HE, Morse MA, Hsu SD (2011). A phase II trial of bevacizumab plus everolimus for patients with refractory metastatic colorectal cancer. Oncologist.

[27] Bendell JC, Jones SF, Hart L, Spigel DR, Lane CM, Earwood C (2015). A phase Ib study of linsitinib (OSI-906), a dual inhibitor of IGF-1R and IR tyrosine kinase, in combination with everolimus as treatment for patients with refractory metastatic colorectal cancer. Invest N. Drugs.

[28] Wolpin BM, Ng K, Zhu AX, Abrams T, Enzinger PC, McCleary NJ (2013). Multicenter phase II study of tivozanib (AV-951) and everolimus (RAD001) for patients with refractory, metastatic colorectal cancer. Oncologist.

[29] Schwark WS, Haluska M (1987). Prophylaxis of amygdala kindling-induced epileptogenesis: comparison of a GABA uptake inhibitor and diazepam. Epilepsy Res.

[30] Baum M (1968). A clinical trial of mithramycin in the treatment of advanced malignant disease. Br J Cancer.

[31] Quarni W, Dutta R, Green R, Katiri S, Patel B, Mohapatra SS (2019). Mithramycin A inhibits colorectal cancer growth by targeting cancer stem cells. Sci Rep..

[32] Zhao Y, Zhang W, Guo Z, Ma F, Wu Y, Bai Y (2013). Inhibition of the transcription factor Sp1 suppresses colon cancer stem cell growth and induces apoptosis in vitro and in nude mouse xenografts. Oncol Rep..

[33] Bravo-San Pedro JM, Pietrocola F, Sica V, Izzo V, Sauvat A, Kepp O (2017). High-throughput quantification of GFP-LC3(+) dots by automated fluorescence microscopy. Methods Enzymol.

[34] Kepp O, Chen G, Carmona-Gutierrez D, Madeo F, Kroemer G (2020). A discovery platform for the identification of caloric restriction mimetics with broad health-improving effects. Autophagy.

[35] Wang Y, Xie W, Humeau J, Chen G, Liu P, Pol J, et al. Autophagy induction by thiostrepton improves the efficacy of immunogenic chemotherapy. J Immunother Cancer. 2020;8. 10.1136/jitc-2019-000462 PMC7206967 32221018

[36] Bezu L, Sauvat A, Humeau J, Gomes-da-Silva LC, Iribarren K, Forveille S (2018). eIF2alpha phosphorylation is pathognomonic for immunogenic cell death. Cell Death Differ.

[37] Sica V, Maiuri MC, Kroemer G, Galluzzi L (2016). Detection of apoptotic versus autophagic cell death by flow cytometry. Methods Mol Biol.

[38] Metivier D, Dallaporta B, Zamzami N, Larochette N, Susin SA, Marzo I (1998). Cytofluorometric detection of mitochondrial alterations in early CD95/Fas/APO-1-triggered apoptosis of Jurkat T lymphoma cells. Comparison of seven mitochondrion-specific fluorochromes. Immunol Lett.

[39] Cerrato G, Leduc M, Muller K, Liu P, Zhao L, Humeau J, et al. Oleate-induced aggregation of LC3 at the trans-Golgi network is linked to a protein trafficking blockade. Cell Death Differ. 2020;28:1733–52. 10.1038/s41418-020-00699-3 PMC8167183 33335289

[40] Bravo-San Pedro JM, Kepp O, Sauvat A, Rello-Varona S, Kroemer G, Senovilla L (2021). Clonogenic assays to detect cell fate in mitotic catastrophe. Methods Mol Biol.

[41] Rello-Varona S, Lissa D, Shen S, Niso-Santano M, Senovilla L, Marino G (2012). Autophagic removal of micronuclei. Cell Cycle.

[42] Michels J, Vitale I, Senovilla L, Enot DP, Garcia P, Lissa D (2013). Synergistic interaction between cisplatin and PARP inhibitors in non-small cell lung cancer. Cell Cycle.

[43] Humeau J, Sauvat A, Cerrato G, Xie W, Loos F, Iannantuoni F (2020). Inhibition of transcription by dactinomycin reveals a new characteristic of immunogenic cell stress. EMBO Mol Med.

[44] Loos F, Xie W, Sica V, Bravo-San Pedro JM, Souquere S, Pierron G (2019). Artificial tethering of LC3 or p62 to organelles is not sufficient to trigger autophagy. Cell Death Dis.

[45] De Sousa EMF, Wang X, Jansen M, Fessler E, Trinh A, de Rooij LP (2013). Poor-prognosis colon cancer is defined by a molecularly distinct subtype and develops from serrated precursor lesions. Nat Med.

