# Supplementary material for: Everolimus and plicamycin specifically target chemoresistant colorectal cancer cells of the CMS4 subtype
Source: Cell Death Dis. 2021 Oct 21;12(11):978. doi: 10.1038/s41419-021-04270-x (PMC8531384; doi:10.1038/s41419-021-04270-x)
Supplement: Supplementary file 1 — Supplemental Figure Legends [file 41419_2021_4270_MOESM1_ESM.docx]

## Supplemental Figure Legends

**Figure S1. Differential gene expression in MDST8 and LoVo.** MDST8 and LoVo wild type cells were subjected to RNA extraction, followed by the assessment of mRNA expression of target genes by quantitative RT-PCR. Data were normalized to the house keeping gene HPRT1 and are shown as means ± SD of Log2-fold change of three independent experiments. *P* values were calculated by means of the Student’s *t*-test. Only -Log10 *P* values＞2.5 were marked.

**Figure S2. Sunitinib and Crizotinib induce cell death in LoVo**. (**A**) MDST8 or LoVo cells were treated with the 71 drugs of an anticancer drug library at a final concentration of 1 μM and 10 μM for 48 h and 72 h. Cell death was measured by assessing the percentage of AnnexinV^high^DAPI^high^ cells by flow cytometry. Mean values of 3 independent experiments are depicted as a hierarchically clustered heatmap. The tiles in the heat map represent the percentage of AnnexinV^high^DAPI^high^ dead cells in a range from 0 to 100%. (**B**) MDST8 and LoVo cells were treated with sunitinib (SUN, 2 µM, 10 µM and 50 µM), crizotinib (CRIZ, 2 µM, 10 µM and 50 µM) and M2I-1 (2 µM, 10 µM and 50 µM) for 4 days. Apoptosis was measured by flow cytometry upon staining with AnnexinV and DAPI. (**C**) Representative images of cells treated with everolimus (EVE), plicamycin (PLI) or rapamycin (RAPA) at a final concentration of 0.1 μM for 72 h are depicted. Scale bar equals 50 µm.

**Figure S3. Plicamycin induces cell death in Colo320HSR.** Wild type (WT) Colo320HSR and HCT116 cells were treated with plicamycin (PLI at 25, 50 and 100 nM for 72 h), everolimus (EVE at 10, 100 nM and 1 μM for 72 h), oxaliplatin (OXA; 2.5, 5 and 10 μM for 48 h), 5-fluorouracil (5-FU; 2.5, 5 and 10 μM for 48 h), sunitinib (SUN; 2.5, 5 and 10 μM for 48 h). Then, cells were stained with the DAPI and Annexin V to measure apoptotic cell death (**A-C**). (**A**) Representative dot plots of untreated Colo320HSR and HCT116 controls (Ctrl) or treated with plicamycin 100 nM, EVE 1 μM, OXA 10 μM, 5-FU 10 μM and SUN 10 μM. Numbers indicate the percentage of cells in each quadrant. (**B-C**) The frequency of dying (DAPI^low^AnnexinV^high^) and dead (DAPI^high^) cells among the Colo320HSR (**B**) and HCT116 (**C**) cells elicited by the corresponding drugs, as determined by analysis with the Flow Jo software. Data is depicted as mean values of three independent experiments. Error bars indicate SEM. Asterisks refer to significant effects for treatments *versus* control (paired Student’s *t*-test; **P* < 0.05, ***P* < 0.01, ****P* < 0.001).

**Figure S4. MDST8 manifest a loss of mitochondrial membrane potential in response to everolimus and plicamycin.** (**A-F**) MDST8 and Lovo cells were treated with everolimus (EVE; 10 nM; 100nM and 1μM) or plicamycin (PLI; 25, 50 and 100 nM) and incubated for 6 h, 24 h or 48 h. Following cells were stained with MitoTracker™ Orange and Hoechst 33342 and then assessed with automated fluorescence microscopy. Representative images are depicted in **A** (MDST8) and **D** (LoVo), scale bar equals 10 µm. Data from image analysis depicting an alteration in mitochondrial fiber length (**B, E**) or a loss of mitochondrial membrane potential (**C, F**) are depicted as bar charts. Error bars indicate SEM. Asterisks refer to significant effects or treatments *versus* control (paired Student’s *t*-test, **P* < 0.05, ***P* < 0.01, ****P* < 0.001)

**Figure S5. Everolimus and plicamycin mediate improvement of anticancer chemotherapy.** (**A-D**) Five million human colon cancer MDST8 or LoVo cells were injected *subcutaneously* (*s.c.*) into the flank of athymic immunodeficient *nu/nu* mice. Next, tumor size was measured regularly and individual tumor growths of everolimus (EVE) and plicamycin (PLI) versus control (Ctrl) mice are depicted (**A, B**). (**C, D**) Body weight was monitored to reflect drug toxicity according to Figure 6 (**C, D**). *n* ≥ 6 mice per group. Results (means ± SD tumor growth curves) are plotted (ns, not statistically significant *versus* ctrl mice).
