# Supplementary figures and images for: Everolimus and plicamycin specifically target chemoresistant colorectal cancer cells of the CMS4 subtype
Source: Cell Death Dis. 2021 Oct 21;12(11):978. doi: 10.1038/s41419-021-04270-x (PMC8531384; doi:10.1038/s41419-021-04270-x)

# mRNA expression in MDST8 vs LoVo

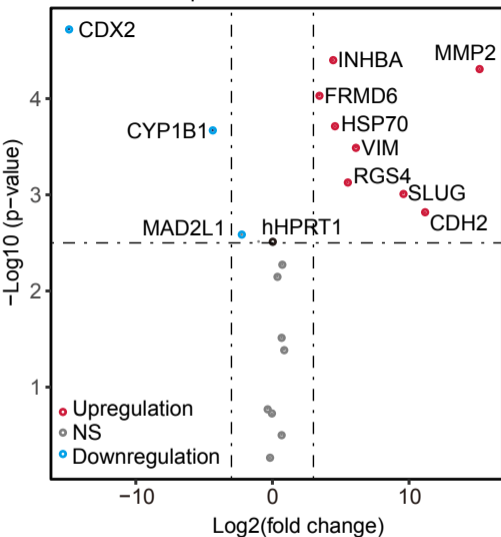

**Figure S1**

Supplement: Supplementary file 2 — Figure S1 [file 41419_2021_4270_MOESM2_ESM.pdf]

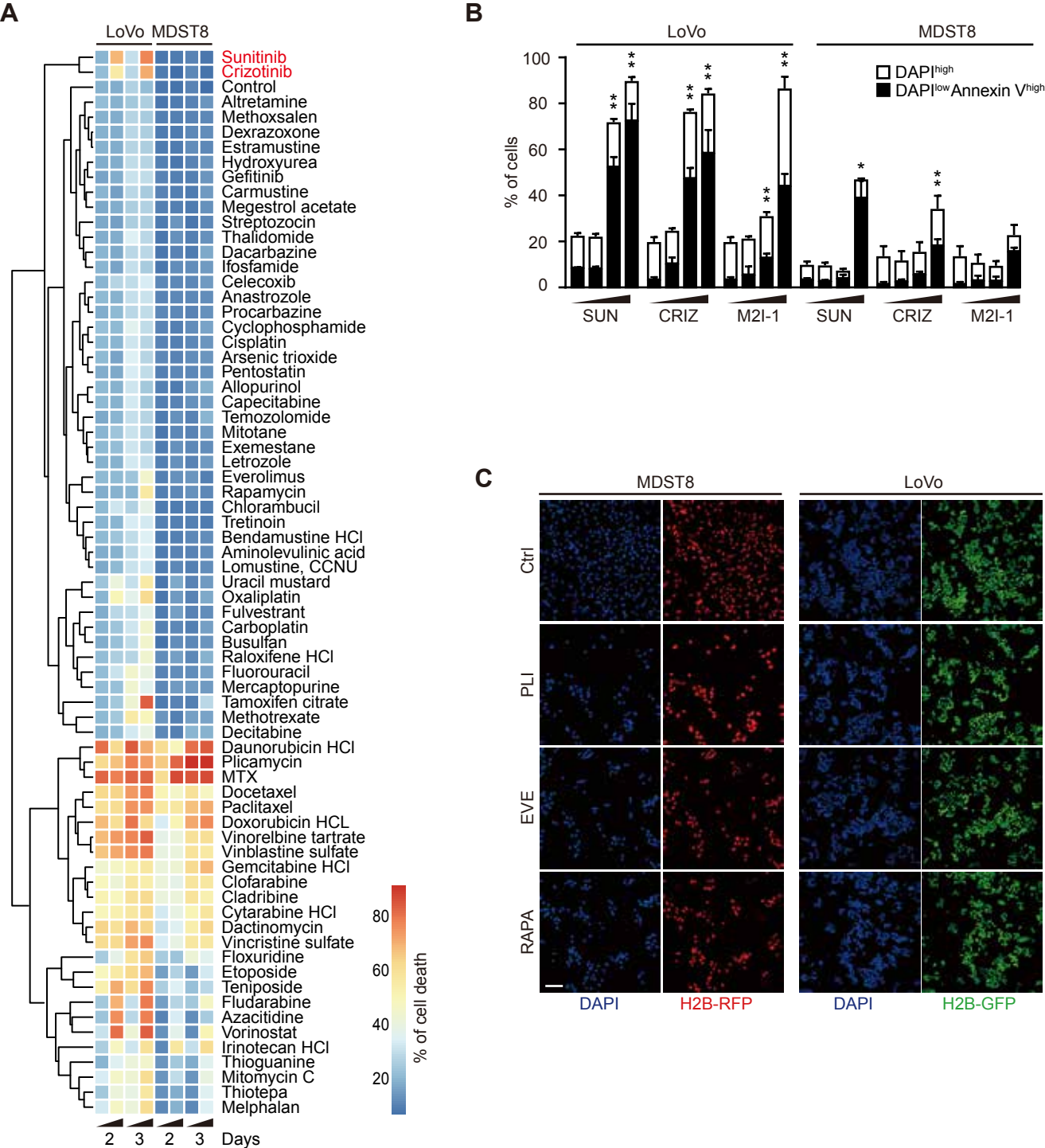

**Figure S2**

Supplement: Supplementary file 3 — Figure S2 [file 41419_2021_4270_MOESM3_ESM.pdf]

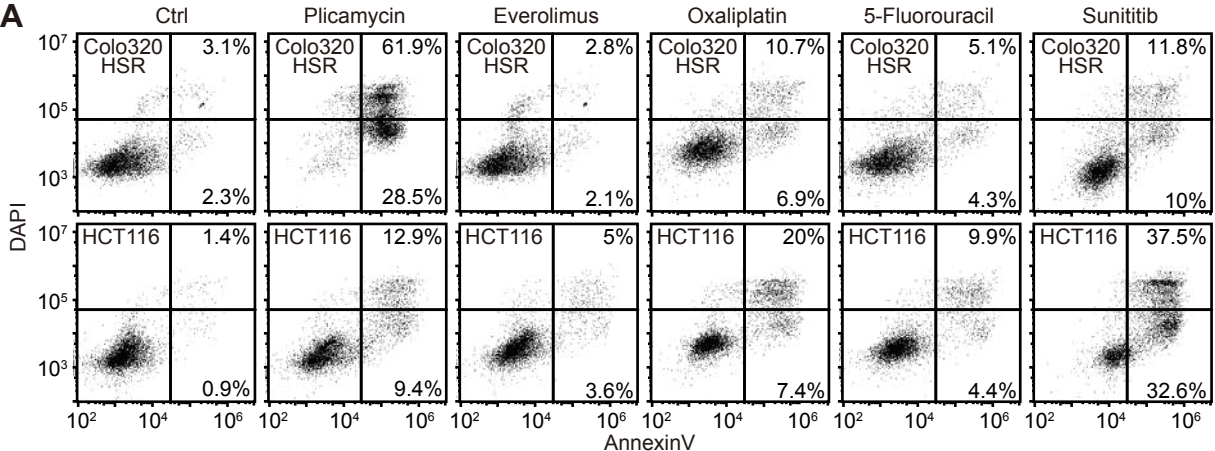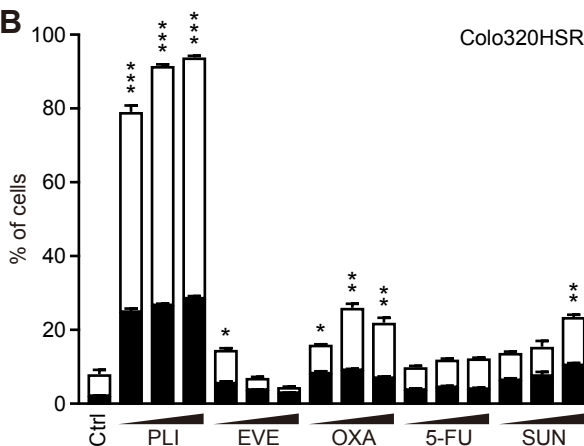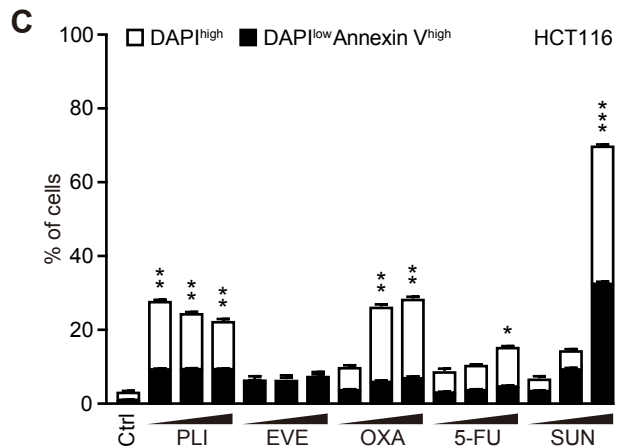

**Figure S3**

Supplement: Supplementary file 4 — Figure S3 [file 41419_2021_4270_MOESM4_ESM.pdf]

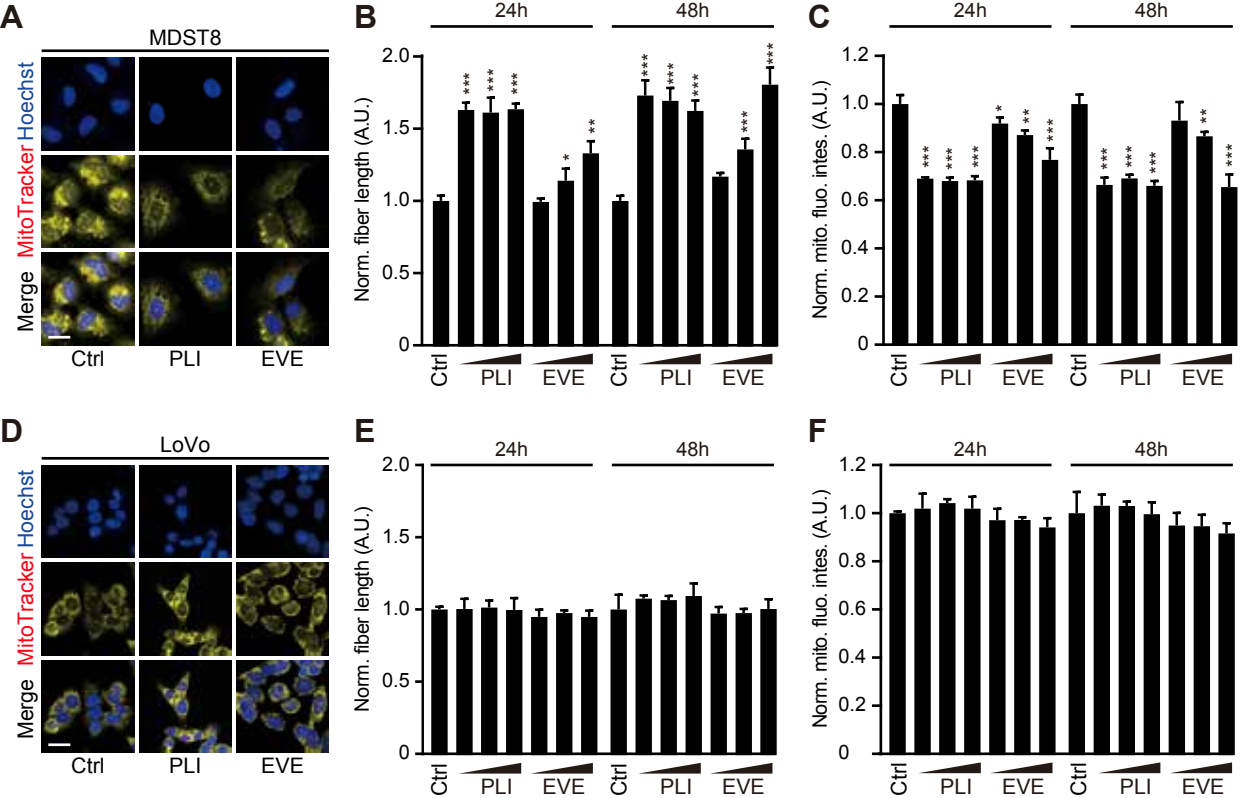

**Figure S4**

Supplement: Supplementary file 5 — Figure S4 [file 41419_2021_4270_MOESM5_ESM.pdf]

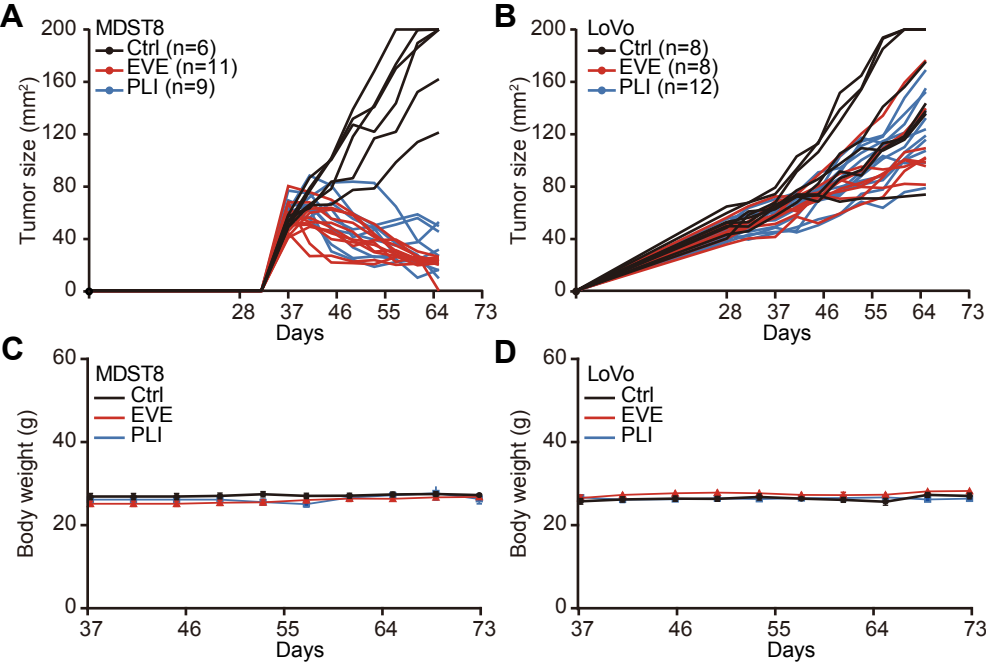

**Figure S5**

Supplement: Supplementary file 6 — Figure S5 [file 41419_2021_4270_MOESM6_ESM.pdf]
